# Effects of unilateral dynamic handgrip on reaction time and error rate

**DOI:** 10.1007/s10339-022-01080-7

**Published:** 2022-02-10

**Authors:** Arash Mirifar, Mengkai Luan, Felix Ehrlenspiel

**Affiliations:** 1 Department of Sport and Health Sciences, Faculty of Sports and Health Sciences, Chair of Sport Psychology, Technical University of Munich, Uptown Munich, Campus D - Georg-Brauchle-Ring 60/62, 80992 Munich, Germany; 2 Institute of Sports Science, Leibniz University Hannover, Hannover, Germany; 3 Department of Psychology, Shanghai University of Sport, 650 Qing Yuan Huan Road, Shanghai, 200438 People’s Republic of China

**Keywords:** Dynamic handgrip, Muscle contractions, Perceptual-motor ability, Reaction time

## Abstract

Quick and accurate reactions to environmental stimuli are often required. Researchers have investigated ways to improve these reactions, which are critical components of perceptual-motor abilities. To optimize individual performance, different techniques, such as embodied interventions and brain stimulation, have been examined. The evidence from EEG studies shows that upper limb muscle contractions lead to changes in brain oscillations associated with changes in mental states and behavioral outcomes. Much research has been conducted on whether muscle contractions of a particular hand have a greater effect on a perceptual-motor ability, as a trigger to facilitate cortical processes (a mediator) for skilled motor performance. While previous studies have shown that left- (vs. right-) hand contractions can lead to greater alpha activation, we hypothesized that left dynamic handgrips have different impacts on motor performance, reflected by simple RT (SRT) and choice RT (CRT). We recruited 64 right-handers, for a within/between-subjects experiment consisting of performance measurements in SRT and CRT tasks after the intervention (either right or left dynamic handgrip approximately twice a second for 30 s for each hand) or assignment to paired passive control groups. We did not find left-hand contractions improve response accuracy in neither SRT nor CRT tasks. Further, left-hand contractions did not affect RTs. The findings indicate that the effects of dynamic handgrips are smaller on behavioral outcomes such as RTs than what can be inferred from published studies. More research is needed to establish the effect of dynamic handgrips on optimizing performance.

**Supplementary Information:**

The online version contains supplementary material available at 10.1007/s10339-022-01080-7.

## Introduction

In many domains, individuals frequently encounter situations that require efficient processing of environmental stimuli and speedy responses. An individual’s quick and accurate reaction to stimuli is therefore a critical aspect of their perceptual-motor abilities. As a result, researchers have been looking for ways to improve reaction times using different techniques, ranging from simple cognitive training (see e.g., Simpson et al. 2012) to more complicated approaches such as brain stimulation (see e.g., Angelakis et al. 2007), to treat impairments in these abilities caused by mental disorders and/or to optimize individual performance (see e.g., Hashemian et al. 2013; Hatfield et al. 2009; Jeunet et al. 2019). Embodied interventions—bodily actions intended to change cortical activity in order to change behaviors (Beckmann et al. 2013; Mirifar et al. 2020)—have also attracted attention in this area because they offer a practical, easily accessible, and affordable approach. One such simple but effective embodied intervention is dynamic handgrip; however, its effect on reaction times (RTs) has not yet been investigated.

Behavioral studies have provided evidence that unilateral upper limb muscle contractions have positive effects on, among others, motivated behavior (Harmon-Jones 2006), affect and emotion (Peterson et al. 2008; Propper et al. 2017), and creative thinking (Goldstein et al. 2010). In the field of sports, a series of experiments by Beckmann et al. (2013) showed that performance under pressure of self-paced motor skills improved only after dynamic left-hand grip.

Unilateral upper limb muscle contractions lead to EEG oscillations in contralateral hemispheres of the brain, predominantly in the form of an increase in the power/amplitude of alpha waves (8–13 Hz; Harmon-Jones 2006; Hirao and Masaki 2018). This phenomenon was mostly tested during the contraction phase and, based on the behavioral outcomes, was usually assumed to persist after termination of contractions (see e.g., Harmon-Jones 2006). However, no secondary resting states had been assessed post-contraction, until Cross-Villasana et al. (2015) and Mirifar et al. (2020), who tested the neurophysiological after effects of such unilateral hand contractions. These studies showed a long lasting reduction in cortical activity (indicated by an increase in the power/amplitude of alpha waves) after unilateral upper limb contractions, a change which was especially evident when the left hand was dynamically contracted. This has been termed left-hand dynamic handgrip or LDH. The data from these studies also showed that a depression of cortical excitability (i.e., increase power in alpha waves) in both hemispheres after the left-hand (but not the right hand) intervention was terminated (Cross-Villasana et al. 2015; Mirifar et al. 2020). Thus, LDH appeared to generate a relaxation effect that can reduce or eliminate performance deterioration in motor tasks under pressure (Beckmann et al. 2013; Gröpel and Beckmann 2017).

An increase in the power of brain activity in the range of 8–15 Hz is generally recognized as a state of (relaxed) readiness for processing information and preparing for motor action (Egner and Gruzelier 2004; Taylor and Thut 2012; Wyrwicka and Sterman 1968). Therefore, there may be a link between increased alpha wave power, better mental processing, and consequently, faster RTs. In line with this hypothesis, empirical evidence has shown that older people respond to stimuli more slowly than younger people, and this phenomenon has been linked to a reduction in alpha wave power in older people’s electroencephalogram (EEG; Gajewski and Falkenstein 2014; Porciatti et al. 1999; Roubicek 1977). In contrast, studies in the field of neurofeedback training (NFT) have shown that increasing alpha wave power fails to reduce RTs, and in fact, that decreasing alpha wave power led to significantly faster RTs (Woodruff 1975). In line with this finding, increasing the power/amplitude of faster brain frequencies, such as sensory-motor rhythm (SMR 12–15 Hz, Doppelmayr and Weber 2011), or a combination of decreasing slow waves (such as theta waves in the range of 4–8 Hz) and increasing fast waves (such as beta waves in the range of 13–30 Hz), tends to lead to faster RTs (Egner and Gruzelier 2004). These changes in the response time were observed in both simple (SRT) and choice reaction time (CRT) tasks; however, inconsistencies were reported when the training frequency differed. RT is the most widely used measure in neuroscience and psychology for noninvasively assessing brain processing of a stimulus; furthermore, RT is speculated to reflect the time needed to complete the perceptual and motor-planning computations required to prepare a response (Wong et al. 2017). Previous research has established that response time can variate due to latency variations in different brain regions, e.g., areas involved in sensory perception, sensory-motor transformation, or motor execution (Rizzolatti and Luppino 2001; Schall 2003; Sternberg 1969) due to, e.g., differences in the components of RT tasks. Several lines of evidence suggest that unilateral hand contractions can influence some cognitive components underlying RT, such as working memory (e.g., episodic recall, Andreau and Torres Batan 2019; Propper et al. 2013; and semantic processing, Turner et al. 2017) and global/local attentional processing (Gable et al. 2013).

The current study addresses the following questions: (a) do unilateral upper limb muscle contractions have an effect on RT, and if yes, how do these effects differ between hands; and (b) which types of RT are affected and in what way? An SRT task typically creates a situation in which only one type of response follows a given stimulus. In contrast, a CRT task is characterized by at least two different responses mapped onto different stimuli. Each stimulus is associated with a specific response, and participants must select the correct response to the given stimulus. This additional stage of processing goes beyond what is required for an SRT task. As other parameters, such as motor and perceptual speed, are considered identical for both SRT and CRT tasks, comparisons between these two types of tasks enable assessment of the underlying internal motor-cognitive processes, notably attentive sensory-motor mapping and response selection (Ives 2013). A shorter time, therefore, is required to respond to a stimulus in a SRT task and a longer time in a CRT task.

From a neurophysiological perspective, it is known that a faster response time is associated with faster EEG oscillatory activity and an increased level of arousal, but this association may not necessarily lead to a more accurate response. In fact, this association has been connected with increased levels of arousal induced by activation of the noradrenergic network of alertness/vigilance and attention (Posner and Petersen 1990; Posner and Raichle 1994). Therefore, we hypothesize that LDH will have different effects on SRT and CRT. This hypothesis is based on the fact that the LDH has been shown to increase alpha power across the whole cortex (a finding that has been robustly shown in several EEG studies, e.g., Cross-Villasana et al. 2015; Hirao and Masaki 2018; Mirifar et al. 2020), which might influence perception and the underlying internal motor-cognitive processes—notably attentive sensory-motor mapping and response selection—in response time tasks. An accumulating body of data suggests that inter-areal alpha-phase synchronization could support attentional, executive, and contextual functions (Palva and Palva 2011). Further, we hypothesize that such increases in alpha power induced by LDH will improve accuracy in CRT tasks (indicated by fewer omission and commission errors), though these increases in alpha power will slow response times, specifically, in SRT due to a decline of arousal.

## Methods and materials

### Participants

Following the sample selection criteria used in previous studies (Cross-Villasana et al. 2015; Mirifar et al. 2020), we included participants between 18 and 30 years of age, who were right-handed according to a laterality quotient of + 50 or higher of the Edinburgh Handedness Inventory (Oldfield 1971). We conducted a prior power analysis using G*Power (Faul et al. 2007) to estimate the required participant sample size. Based on the effect size reported in Mirifar et al. (Mirifar et al. 2020; Cohen’s *f*: 0.55), an alpha level of 0.05, a power level of 0.8 and correlations among repeated measurements 0.5, a sample size of 11 participants for each of our four groups is sufficient. Mirifar et al. (2020), however, only showed the difference in the electroencephalographic alpha amplitude. Whether the difference in RTs might show a similar magnitude was not known before the current study. Sixty-six healthy undergraduate and graduate students were recruited for this study. They had a mean age of 24.30 years (SD 3.8), and their mean laterality quotient on the Edinberg Handedness Test (Oldfield 1971) was + 79.39 (range: + 58.33 to + 100). Participants were randomly assigned to one of the four following groups: (a) left dynamic handgrip (LDH, 16 participants), (b) right dynamic handgrip (RDH, 17 participants), (c) control group paired with left dynamic handgrip (C-LDH, 17 participants), and (d) control group paired with right dynamic handgrip (C-RDH, 16 participants). One participant in the LDH group was excluded from the analysis because of a misunderstanding of the instructions. And one participant in the RDH group was excluded from the further analysis because of high error rates in the CRT task (larger than 35%). Thus, our final cohort consisted of 64 right-handed participants, of which 29 were female and 35 were male. With this sample, Cohen’s *f* higher than 0.44 can be detected with a power level of 0.8, an alpha level of 0.05 and correlations among repeated measurements 0.5 by a sensitivity analysis using G*Power (Faul et al. 2007).

After screening prospective participants for age and apparent handedness, we invited them individually to take part in the experiment. We explained the experimental procedures and purpose of the study, the rights of the participant, and processes to ensure data anonymity. Afterward, they were invited to join the study by providing signed informed consent, according to the Declaration of Helsinki. After informed consent, participants completed the Edinburgh Handedness Inventory (Oldfield 1971), and then received instructions on how to execute a dynamic handgrip by giving a demonstration of its execution. All participants were then asked to demonstrate the correct execution prior to task participation. The study did not involve any invasive or potentially dangerous methods and therefore, in accordance with the German Research Foundation (DFG) and the guidelines of the first author’s institution, did not require formal ethical approval.

### Procedure

**Fig. 1:**
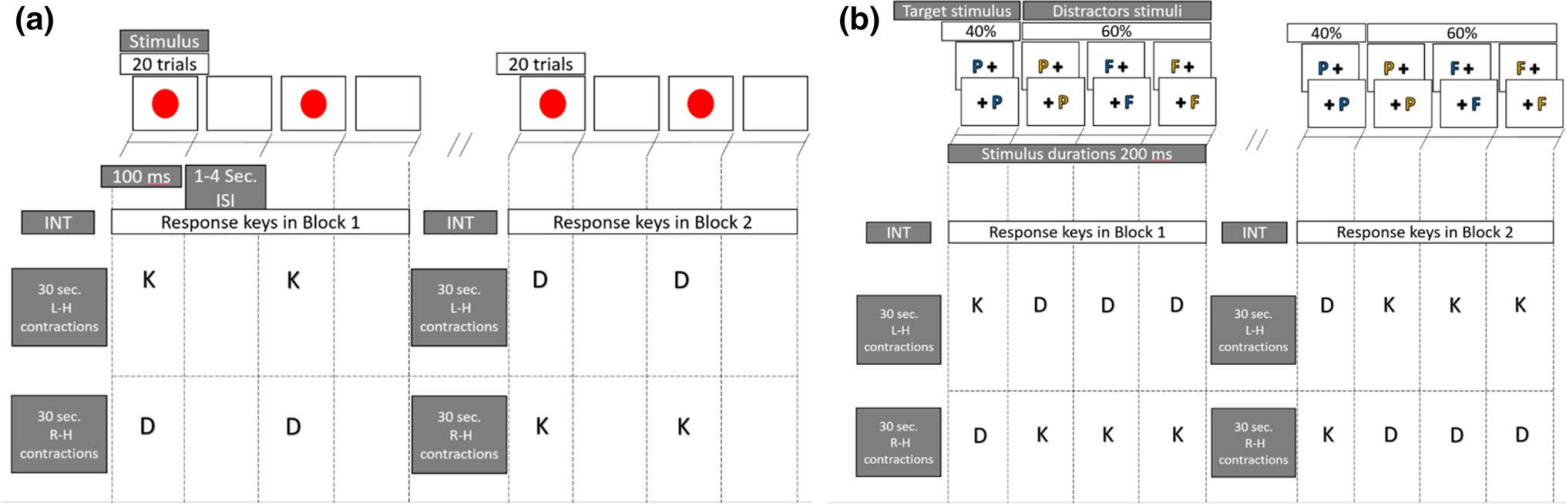
Schematic of the experimental procedure and tasks. Note: **A** SRT task and blocks; **B** CRT task and blocks. INT stands for intervention. In the CRT task, the colored letters (blue P, orange P, blue F, or orange F) were randomly presented, subtending 0.5° of visual angle, to the left or right hemifield, 1.6° from the fixation cross (+)

The experiment consisted of three phases: (a) pretest, (b) intervention, and (c) test. The pretest was conducted to examine if there is a difference between the intervention groups with their paired control groups in perceptual-motor ability (SRT and CRT) before the intervention phase. Each participant completed a 10-trial SRT task and a 20-trial CRT task (details described below). The results of independent samples t tests showed that both reaction times and error rates of the intervention groups did not differ from those of their paired control groups (*p*s < 0.05). That means participants in different groups were initially similar in perceptual-motor ability. (This analysis displayed by figures in the supplementary materials named S1.) The main body of the experiment was divided into two blocks; each of which consisted of an intervention phase and a test phase. Participants from the intervention groups (i.e., left or right dynamic handgrip) underwent their respective dynamic handgrip, whereas those from the control groups rested silently and were immobile during this phase. The test phase consisted of the SRT task first, followed by the CRT task. In the SRT task, participants were asked to respond to target stimuli with the required hand depending on the block. In the CRT task, participants were asked to respond to target and distracting stimuli with different hands depending on the block; for instance, either with the ipsilateral or contralateral hand in reference to the intervention side (i.e., hand squeezing). With regard to fatigue of the human motor system (which e.g., may be caused by repetitive hand contractions), as a moderator in response time performance, Soto-Leon et al. (2020) have shown that fatiguing tasks impair response times. The benefit of our approach (switching hands in response to target stimuli depending on the block) is that we can control the effects of fatigue of the human motor system on the simple and choice reaction time performance. Our study design is shown in Fig. 1.

### Intervention

We used dynamic handgrip as the intervention in these experiments. It consisted of holding a soft rubber ball (6 cm in diameter) in either the left or right hand depending on group allocation, and repeatedly squeezing it completely with all fingers for a period of 30 s (adapted from Beckmann et al. 2013). Participants were instructed to squeeze and release the ball at their own pace, but to maintain an approximate rate of two squeezes per second. The other hand was kept on the matching thigh with the palm facing down.

### RT tasks and response conditions

RT tasks were programmed and implemented in MATLAB (MathWorks Inc., Natick, MA, USA) and Psychtoolbox extensions (http://psychtoolbox.org/).

#### SRT

In the SRT task, 40 stimuli in each block were presented, using a mean interstimulus interval (ISI) of 2.5 s, which was derived from a rectangular distribution with a minimum of 1 s and a maximum of 4 s. The stimulus in the SRT task was a red circle (2° visual angle) shown on a gray background. Participants responded to the stimulus in the first block by pressing either the “K” key with the right index finger or the “D” key with the left index finger, following left- and right-hand contractions, respectively. The response keys were switched for the second block. Stimulus durations were fixed at 100 ms. An error of omission was calculated if participants failed to perform a necessary step or action to respond to target items within 1500 ms. Task performance for each block was indexed by the mean of correct responses from all 40 response times and presented as SRT. (The analysis based on the median is provided in the supplementary materials named S2.) Each block of the simple RT task required ~ 2 min.

#### CRT

The CRT task was adapted from previous studies (Mirifar et al. 2019; Woods et al. 2015), and consisted of two types of stimuli: target (the letter “P” in blue font, “blue P”) and distractor (“orange P,” “blue F,” or “orange F”). A total of 100 stimuli were presented, of which 40 (40%) were target stimuli. Stimuli were presented on the same gray background as the SRT task. In the first block, participants from the LDH group responded to target stimuli with the “K” key and to distracting stimuli with the “D” key, using the right or left index finger, respectively. The response keys for target and distracting stimuli were then switched in the second block. In contrast, in the first block, participants from the RDH group responded to target stimuli with the “D” key and to distracting stimuli with the “K” key, using the respective left or right index finger. Similar to the LDH group, the response keys for target and distracting stimuli were switched in the second block. This switch was also implemented for both of the paired control groups in the second block. All stimulus durations were fixed at 200 ms. To make the CRT task challenging, ISI began at 2.5 s (see Mirifar et al. 2019), and it was adapted as a function of accuracy. Two consecutive correct responses (hits) resulted in a 3% ISI decrease, whereas each error, miss, or nonresponse resulted in a 3% ISI increase. Performance in the CRT task was indexed by the mean of correct responses of the 40 target response times and presented as CRT. (The analysis based on the median is provided in the supplementary materials named S2.) Each block of the choice RT required ~ 6 min. (To control for the potential effects of the intervention within this ~ 6 min, the analysis of the performance in the first 40 trials of each block in the CRT task is provided in the supplementary materials named S3.)

### Edinburgh handedness inventory

Handedness was assessed using a revised version of the Edinburgh Handedness Inventory (Oldfield 1971). Participants were asked to indicate their hand preference for each listed activity by marking a cross (+) in the appropriate column. Scores are continuous and calculated as a percentile of handedness, with laterality coefficients ranging from − 100 (completely left-handed) to + 100 (completely right-handed). A person with a laterality quotient + 50 or higher is considered right-handed (Oldfield 1971).

### Data analyses

For the SRT task, trials with latencies exceeding 1500 ms were counted as missing, and SRTs faster than 100 ms were considered to be anticipated. Trials with response omission and anticipation were excluded from analyses of SRTs. For each intervention-control pair, mean SRTs and error rates (excluding anticipation trials) computed for each factor combination were analyzed using mixed 2 (group: intervention, control) × 2 (response hand: left, right) ANOVAs with group as the between-subject variable.

For the CRT task, trials with latencies exceeding 1500 ms were counted as missing, and CRTs faster than 250 ms were considered to be anticipated. The anticipation threshold was adapted from previous studies (this setting was adapted from a previous studies, Mirifar et al. 2019; Woods et al. 2015), and wrong keypress, response omission, and anticipation were excluded from analyses of CRTs. For each intervention-control pair, mean CRTs and error rates (excluding anticipation trials) computed for each factor combination were analyzed using mixed 2 (group: intervention, control) × 2 (response hand: left, right) ANOVAs with group as the between-subject variable.

## Results

### SRT task

**Fig. 2:**
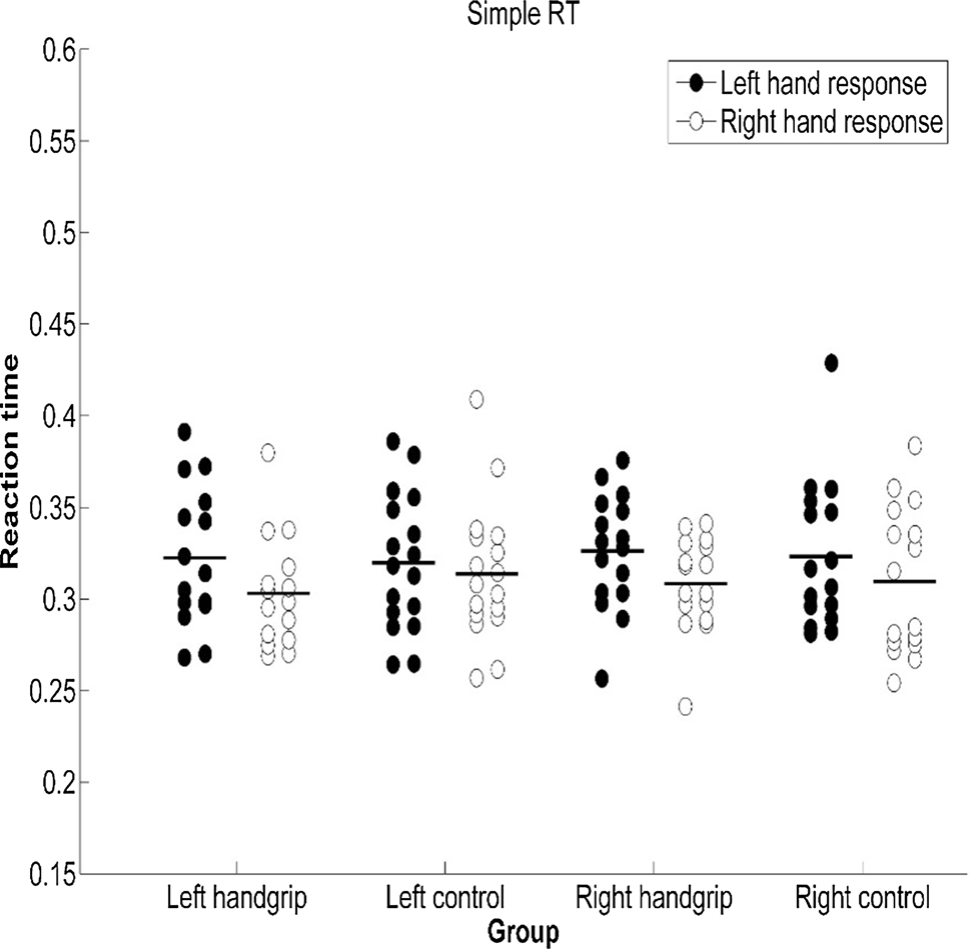
SRT of groups when the SRT task was executed under two different conditions. Note: Participants were asked to respond to target stimuli with the required hand, depending on the block, either with the ipsilateral or contralateral hand with reference to the intervention side (i.e., hand squeezing). Error bars represent standard errors

**Fig. 3:**
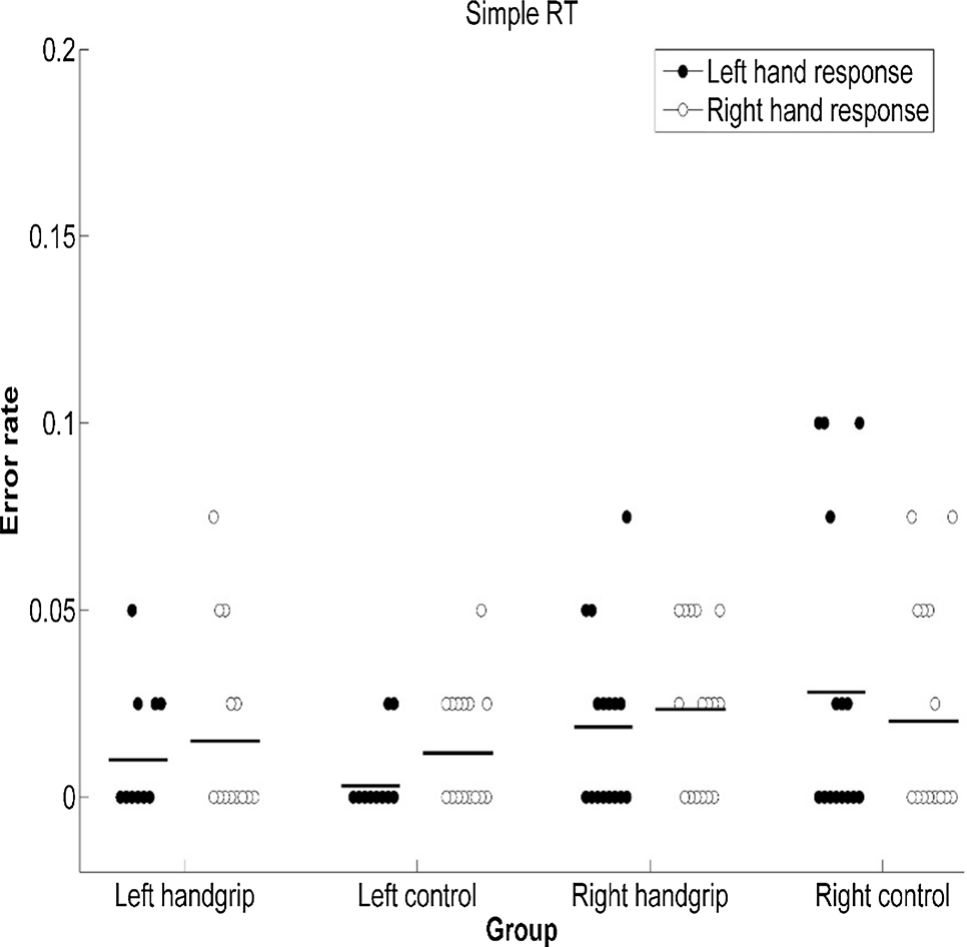
Error rate of groups when the SRT task was executed under two different conditions. Note. Participants were asked to respond to target stimuli with the required hand, depending on the block, either with the ipsilateral or contralateral hand with reference to the intervention side (i.e., hand squeezing). Error bars represent standard errors

Based on ANOVA of SRTs of the left handgrip-control pair, there was no significant difference in SRT performance between the left handgrip group and the left control group [*F*(1, 30) = 0.01, *p* = 0.91, ${\eta}_{p}^{2}$ < 0.001], which was inconsistent with our expectations. We observed a significant difference in SRT between right-hand response and left-hand response [*F*(1, 30) = 6.35, *p* = 0.017, ${\eta}_{p}^{2}$ = 0.18]; the right-hand response (*M* = 308 ms) was faster than the left-hand response (*M* = 321 ms). And there was no significant interaction between group and response hand [*F*(1, 30) = 0.95, *p* = 0.34, ${\eta}_{p}^{2}$ = 0.03]. In addition, we found that response errors were infrequent in both the intervention and control group (1.3% and 0.7%, respectively), and ANOVA of error rates did not produce any effect, [*F*s < 2.41, *p*s > 0.13, ${\eta}_{p}^{2}$ s < 0.07; see Figs. 2, 3].

Similar results were found for the right handgrip-control pair. ANOVA of SRTs showed there was no significant difference in SRT performance between the right handgrip group and the right control group [*F*(1, 30) = 0.1, *p* = 0.94, ${\eta}_{p}^{2}$ < 0.001]. We observed a significant difference in SRT between right-hand response and left-hand response [*F*(1, 30) = 20.85, *p* < 0.001, ${\eta}_{p}^{2}$ = 0.41]; the right-hand response (*M* = 309 ms) was faster than the left-hand response (*M* = 325 ms). And there was no significant interaction between group and response hand [*F*(1, 30) = 0.34, *p* = 0.57, ${\eta}_{p}^{2}$ = 0.01]. Response errors were infrequent (2.1% and 2.4% in the intervention and control group, respectively), and ANOVA of error rates did not produce any effect [*F*s < 1.51, *p*s > 0.23, ${\eta}_{{\varvec{p}}}^{2}$ s < 0.05; see Figs. 2, 3].

### CRT task

**Fig. 4:**
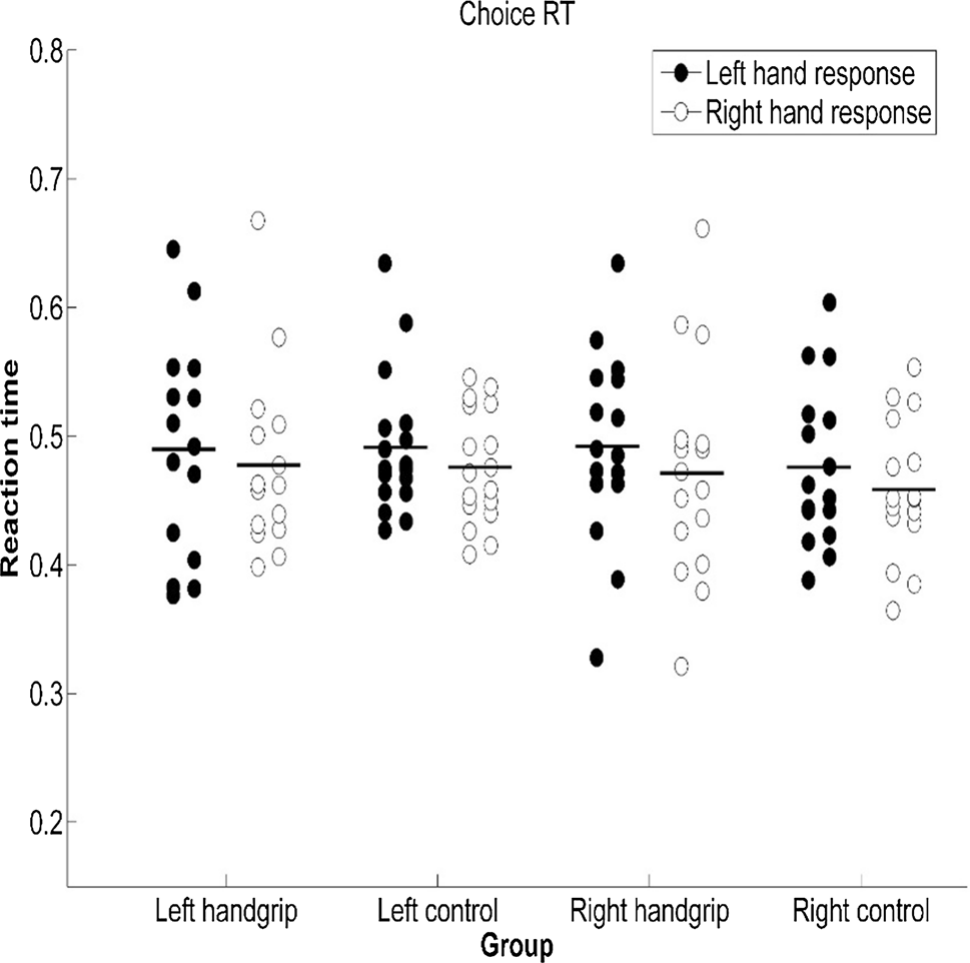
CRT of groups when the CRT task was executed under two different conditions. Note: Participants were asked to respond to target stimuli and distracting stimuli with the required hand, which was dependent on the block, either with the ipsilateral or contralateral hand with reference to the intervention side (i.e., hand squeezing). Error bars represent standard errors

**Fig. 5:**
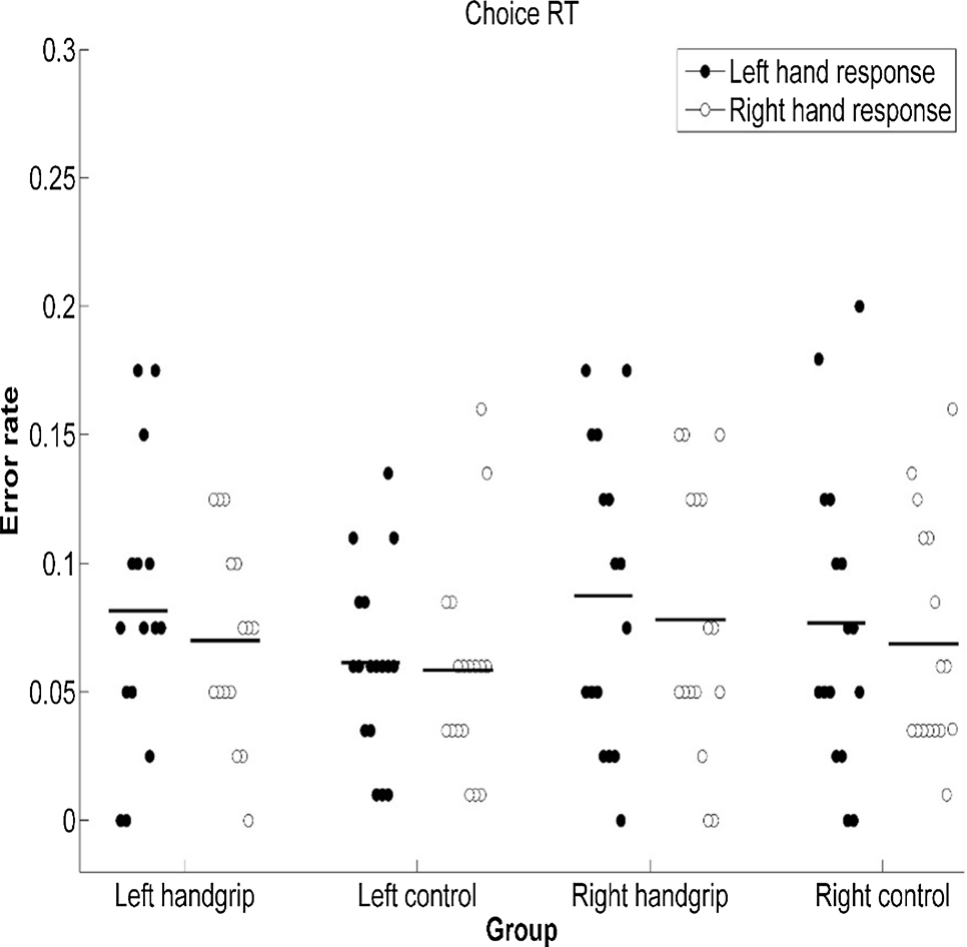
Error rate of groups when the CRT task was executed under two different conditions. Note: Participants were asked to respond to target stimuli and distracting stimuli with the required hand, which was dependent on the block, either with the ipsilateral or contralateral hand with reference to the intervention side (i.e., hand squeezing). Error bars represent standard errors

Next, we evaluated performance on the CRT task. For the left handgrip-control pair, from ANOVA of CRTs, we did not observe any significant difference in CRT between the left handgrip group and the left control group [*F*(1, 30) < 0.001, *p* = 0.97, ${\eta}_{p}^{2}$ < 0.001], or between right-hand response and left-hand response [*F*(1, 30) = 2.53, *p* = 0.12, ${\eta}_{p}^{2}$ = 0.08]. There was also no significant interaction between group and response hand [*F*(1, 30) = 0.04, *p* = 0.85, ${\eta}_{p}^{2}$ = 0.01]. In addition, we found that response errors were infrequent in both the intervention and control group (7.6% and 6.0%, respectively), and ANOVA of error rates did not produce any effect, [*F*s < 1.67, *p*s > 0.21, ${\eta}_{p}^{2}$ s < 0.05; see Figs. 4, 5].

Based on ANOVA of CRTs of the right handgrip-control pair, we found that there was no significant difference in CRT between the right handgrip group and the right control group [*F*(1, 30) = 0.37, *p* = 0.55, ${\eta}_{{\varvec{p}}}^{2}$ = 0.01]. There was also no significant interaction between group and response hand [*F*(1, 30) = 0.06, *p* = 0.81, ${\eta}_{{\varvec{p}}}^{2}$ = 0.002]. We observed a significant difference in CRT between right-hand response and left-hand response [*F*(1, 30) = 7.44, *p* = 0.011, ${\eta}_{{\varvec{p}}}^{2}$ = 0.20]; the right-hand response (*M* = 465 ms) was faster than the left-hand response (*M* = 484 ms). Response errors were infrequent (8.3% and 7.3% in the intervention and control group, respectively), and ANOVA of error rates did not produce any effect [*F*s < 1.80, *p*s > 0.19, ${\eta}_{{\varvec{p}}}^{2}$ s < 0.06; see Figs. 4, 5].

## Discussion

In this study, we investigated the effects of dynamic handgrip on perceptual-motor task performance by assessing speed (RT) and accuracy (error rate) of the response to stimuli in SRT and CRT tasks in the final cohort of 64 right-handed participants. Previous neural and behavioral studies found prevention of performance decrements or even increases in performance on motor tasks with dynamic handgrip (Beckmann et al. 2013; Hoskens et al. 2020). However, RTs and error rates in perceptual-motor tasks have not yet been addressed with this embodiment technique. We hypothesized that participants making left-hand contractions would show improved response accuracy (as behavioral after effects) when performing SRT and CRT tasks, which would be indicated by fewer omission and commission errors, compared with the participants in the passive control groups, who would show no improved response accuracy during tasks. In the same line, we did not expect to see any changes in the right-hand contraction group, as this group was supposed to only play the role of an active control group. Moreover, we hypothesized that making left-hand contractions would lead to slower RTs, whereas participants engaged in right-hand contractions, and those from the passive control groups would show no changes in response time. Contrary to expectations, LDH was not found to improve response accuracy in neither the SRT nor CRT task. Furthermore, left-hand contractions did not lead to slower RTs.

We based our hypotheses on previous findings showing both neurophysiological effects (i.e., increased alpha power, Cross-Villasana et al. 2015; Mirifar et al. 2020) and behavioral effects of dynamic handgrip (e.g., Beckmann et al. 2013; Gröpel and Beckmann 2017). More specifically, using this approach, researchers have been able to show positive effects on some cognitive components underlying RT, such as working memory (e.g., episodic recall, Andreau and Torres Batan 2019; Propper et al. 2013, and semantic processing; Turner et al. 2017) and global/local attentional processing (Gable et al. 2013). However, recent studies also found no effects of left-hand contraction. As a pre-performance routine, dynamic handgrip did not lead to greater accuracy in a beach volleyball service under pressure (Wergin et al. 2020). Our results are also in accord with those of Stanković and Nešić (2020), who investigated the effects of dynamic handgrip on emotional perception. They did not find any difference in the emotional perception of photographs from either unilateral (intrahemispheric) or bilateral (interhemispheric) hemispheres as a function of hand contraction. Our findings also compare well with those of Hoskens et al. (2020), who reported that contralateral hemisphere activity was revealed for left- versus right-hand contraction conditions. Specifically, left-hand contractions rather than right-hand contractions led to significantly lower T7-Fz connectivity, indicating brain regions involved in conscious engagement in movement control and motor performance during motor planning. However, Hoskens et al. (2020) found no evidence of changes in brain oscillatory activity and neural networks induced by left-hand contractions influenced motor performance (i.e., more accurate performance in the golf-putting task). Further, no changes were found from additional physiological markers, such as electrocardiograms and electromyograms, as well as kinematics. The authors further used mediation analyses to examine whether these markers and kinematics mediated the relationship between hand contractions and golf-putting performance (mean radial error). Although there was no significant difference in performance between the different hand contraction conditions, there was a significant indirect effect from hand squeezing on performance via T7-Fz connectivity (Hoskens et al. 2020). The T7-Fz connectivity mediated the relationship between hand squeezing and motor performance (distance from the target). In a more recent study, however, Hoskens et al. (2021) reported that unilateral hand contractions prior to practicing the golf-putting task did not affect performance differently from the no hand contraction (control) group. The authors even reported hand contractions resulted in worse performance compared to the no hand contraction group during the retention tests. In addition, the performance disrupted in the dual-task transfer in both left- and right-hand contraction groups (Hoskens et al. 2021).

Considering these equivocal results, some moderators or boundary conditions for the effectiveness of dynamic handgrip should be considered. First, the role—either causal or epiphenomenal—of the EEG alpha oscillations for human behavior has been a topic of intense discussion for decades. This might e.g., be due to the fact that there are separable thalamic and cortical alpha pacemakers which become differently active and coupled under different behavioral conditions (Halgren et al. 2019; Saalmann et al. 2012). Previous scalp studies of human traveling alpha waves have also found varying propagation directions and, further, show such alpha waves traveling in different directions traversing distinct cortical hierarchies (Ito et al. 2005; Lozano-Soldevilla and VanRullen 2019). Therefore, a note of caution is due here, as an increase in alpha power through LDH may not necessarily lead to changes in RTs and accuracy in RT tasks. The neurophysiological effect induced by left-hand contractions, additionally, may be small as evidenced by the small effect sizes reported in studies that actually investigated the effects of dynamic handgrip on brain oscillations (see e.g., Cross-Villasana et al. 2015; Mirifar et al. 2020). These small physiological effects are likely not strong enough to lead to significant effects at behavioral levels. In fact, such a discrepancy between neurophysiological and behavioral changes has recently been demonstrated by Tinga et al. (2019), who showed that the effect sizes of neurophysiological outcomes are smaller than those of behavioral outcomes. Another moderator could be the strength of squeezing the ball. Squeezing a ball harder or squeezing a harder ball may lead to a greater reduction in alpha activity during the execution time and consequently, a stronger alpha rebound after the intervention. As in previous studies of neural (Cross-Villasana et al. 2015; Mirifar et al. 2020) and behavioral investigations (Beckmann et al. 2013; Wergin et al. 2020), control of muscle contractions (i.e., applied strength) was limited by the size and resistance of the ball, and by the instructions provided to participants. In our study, we used the same experimental design and instructions to confirm the results of these studies in a new task condition. As the causal role of the strength of muscle contraction in the reduction in alpha activity during execution has recently been confirmed by Hirao and Masaki (2018), the impact of the strength of muscle contractions on subsequent alpha band activity should be incorporated in future studies. Wergin et al. 2020 argued for a further moderator: the experience of “pressure.” It was proposed that pressure induction, such as that caused by simulated competition or the presence of audiences, would help researchers more elegantly detect the effects of intervention (i.e., increase in the power/amplitude of alpha waves) as pressure induction generally increases the level of performer anxiety.

The current study was limited by the absence of neurophysiological data, which in such experiments can be considered a manipulation check. Therefore, because experiments have already shown induced alpha power lasts for at least two minutes (Cross-Villasana et al. 2015; Mirifar et al. 2020), future experiments could add a brief resting period after contractions (such as 30 s) to assess the EEG for after effects, followed by the (cognitive) task of interest to assess effects on the behavioral level. Further, the same experiments would also allow investigation of how the effect of the intervention (i.e., alpha wave power) affects the course of task-related EEG modulations in the preparation for and execution of a physical or cognitive action.

## Conclusions

We were unable to show that contractions of the left-hand lead to a more accurate response during task execution, nor were we able to show that intervention had an impact on response time. In context of the continuing debate regarding the effectiveness of changing brain oscillatory activity and brain circuits on optimizing performance, our results do not support the effectiveness of dynamic handgrip on optimizing behavioral outcomes; therefore, future research needs to be done to determine whether this noninvasive approach is effective for other task conditions. In addition, future studies may benefit from having more varied populations (such as including elderly participants) and more complicated tasks (with different levels of cognitive load).

## Supplementary Information

Below is the link to the electronic supplementary material.

Supplementary file1 (DOCX 127 kb)

Supplementary file2 (DOCX 455 kb)

Supplementary file3 (DOCX 424 kb)

## Declarations

### Open practices statement

Stimulus materials, raw data, and analysis scripts are available on the Open Science Framework (https://osf.io/4d5xs/).
