# Supplementary material for: Effects of unilateral dynamic handgrip on reaction time and error rate
Source: Cogn Process. 2022 Feb 10;23(2):169–78. doi: 10.1007/s10339-022-01080-7 (PMC9072264; doi:10.1007/s10339-022-01080-7)
Supplement: Supplementary file 1 — Supplementary file1 (DOCX 127 kb) [file 10339_2022_1080_MOESM1_ESM.docx]

Supplementary materials 1 for

**Effects of Unilateral Dynamic Handgrip on Reaction Time and Error Rate**

Arash Mirifar*, Mengkai Luan*, and Felix Ehrlenspiel

**SRT Task**

Independent sample *t* tests on the SRTs of the left handgrip-control pair indicated that the SRTs in the left handgrip group did not differ significantly with that in the left control group *t*(30) = 0.30, *p* = .77, *d* = 0.12. Similar results were found for the right handgrip-control pair. Independent sample *t* tests on the SRTs of the right handgrip-control pair indicated that the SRTs in the right handgrip group did not differ significantly with that in the right control group *t*(30) = 0.35, *p* = .73, *d* = 0.14 (See in Figure S1).

Independent sample t tests on the error rates of the left handgrip-control pair indicated that the error rates in the left handgrip group did not differ significantly with that in the left control group *t*(30) = 0.94, p = .35. Similar results were found for the right handgrip-control pair. Independent sample t tests on the error rates of the right handgrip-control pair indicated that the error rates in the right handgrip group did not differ significantly with that in the right control group *t*(30) = 0.59, p = .56, d = 0.23 (See in Table 1).

**CRT Task**

Independent sample *t* tests on the CRTs of the left handgrip-control pair indicated that the CRTs in the left handgrip group did not differ significantly with that in the left control group *t*(30) = 0.79, *p* = .44, *d* = 0.24. Similar results were found for the right handgrip-control pair. Independent sample *t* tests on the CRTs of the right handgrip-control pair indicated that the CRTs in the right handgrip group did not differ significantly with that in the right control group *t*(30) = 0.71, *p* = .48, *d* = 0.24 (See in Figure S2).

Independent sample *t* tests on the error rates of the left handgrip-control pair indicated that the error rates in the left handgrip group did not differ significantly with that in the left control group *t*(30) = 0.30, *p* = .76, *d* = 0.11. Similar results were found for the right handgrip-control pair. Independent sample *t* tests on the error rates of the right handgrip-control pair indicated that the error rates in the right handgrip group did not differ significantly with that in the right control group *t*(30) = 0.34, *p* = .73, *d* = 0.17 (See in Table 2).

Figure S1 SRT of Groups in the pretest


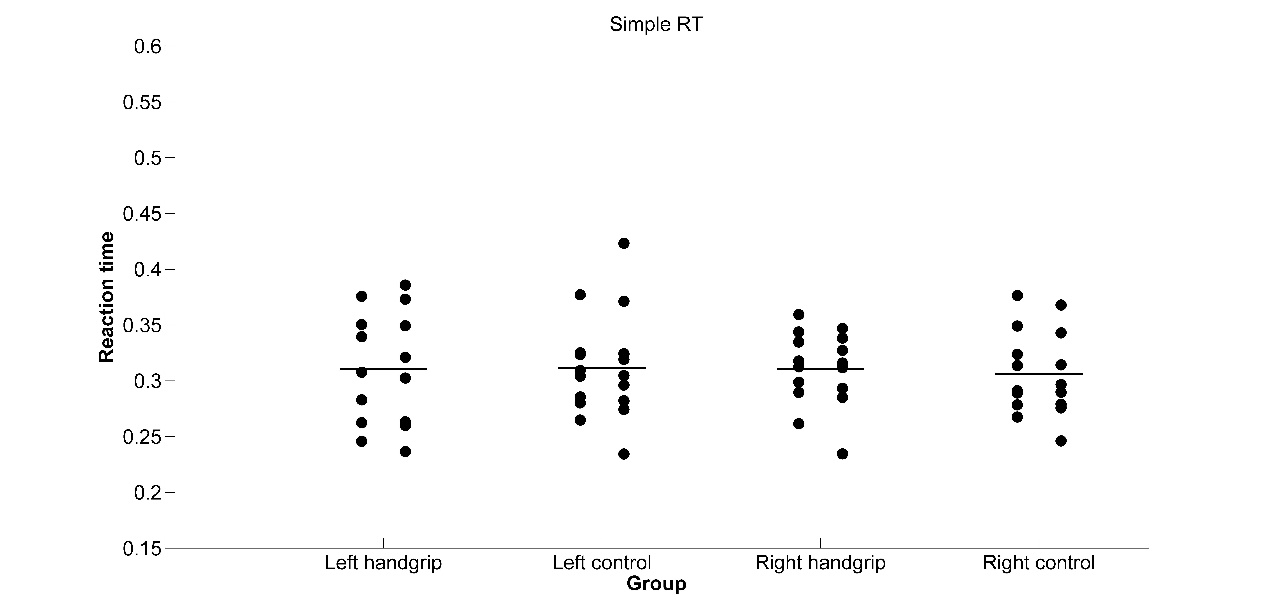


Figure S2 CRT of Groups in the pretest


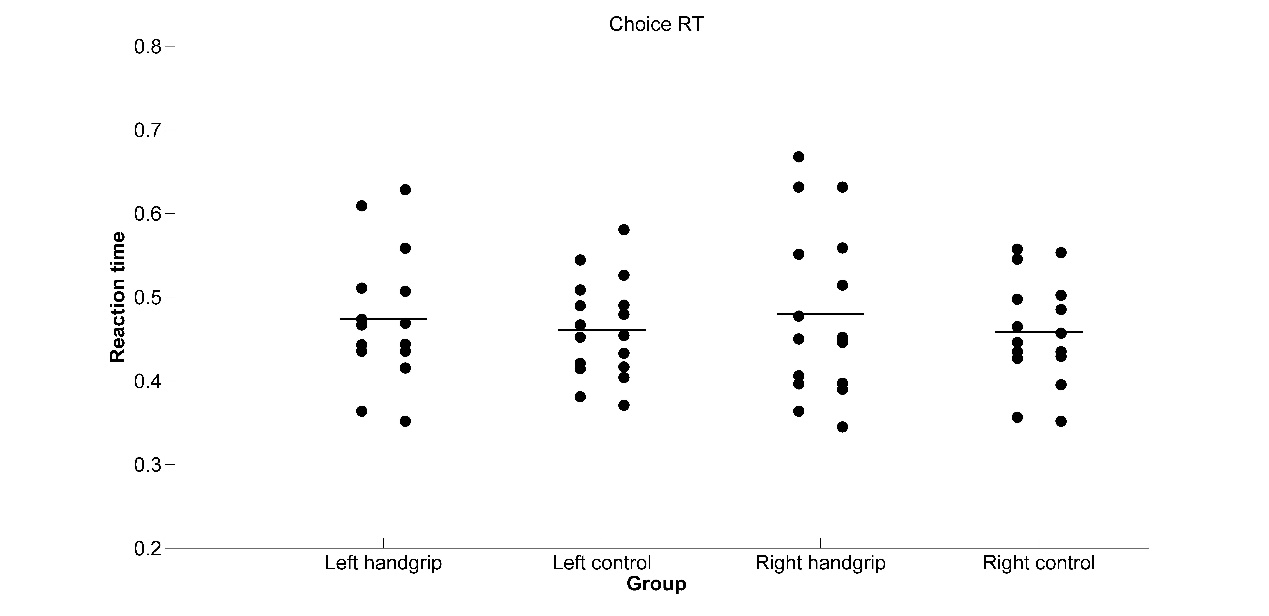


Table S1 Error rates of Groups when the SRT Task was Executed in the pretest

| Group | Mean | SD |
| --- | --- | --- |
| Left handgrip | 0% | 0.00 |
| Left control | 0.6% | 0.02 |
| Right handgrip | 0.6% | 0.03 |
| Right control | 1.3% | 0.03 |

Table S1 Error rates of Groups when the CRT Task was Executed in the pretest

| Group | Mean | SD |
| --- | --- | --- |
| Left handgrip | 5.8% | 0.06 |
| Left control | 5.1% | 0.05 |
| Right handgrip | 7.0% | 0.06 |
| Right control | 6.3% | 0.06 |
