# Supplementary material for: Effects of unilateral dynamic handgrip on reaction time and error rate
Source: Cogn Process. 2022 Feb 10;23(2):169–78. doi: 10.1007/s10339-022-01080-7 (PMC9072264; doi:10.1007/s10339-022-01080-7)
Supplement: Supplementary file 2 — Supplementary file2 (DOCX 455 kb) [file 10339_2022_1080_MOESM2_ESM.docx]

Supplementary materials 2 for

**Effects of Unilateral Dynamic Handgrip on Reaction Time and Error Rate**

Arash Mirifar*, Mengkai Luan*, and Felix Ehrlenspiel

**Results**

**SRT Task**

Based on ANOVA of SRTs of the left handgrip-control pair, there was no significant difference in SRT performance between the left handgrip group and the left control group [*F*(1, 30) = 0.04, *p* = .85, $\text{η}_{p}^{2}$ = .001], which was inconsistent with our expectations. We observed a significant difference in SRT between right hand response and left hand response [*F*(1, 30) = 22.55, *p* < .001, $\text{η}_{p}^{2}$ = .43]. And there was no significant interaction between group and response hand [*F*(1, 30) = 3.37, *p* = .08, $\text{η}_{p}^{2}$ = .10].

Similar results were found for the right handgrip-control pair. ANOVA of SRTs showed there was no significant difference in SRT performance between the right handgrip group and the right control group [*F*(1, 30) = 0.1, *p* = .98, $\text{η}_{p}^{2}$ < .001]. We observed a significant difference in SRT between right hand response and left hand response [*F*(1, 30) = 33.60, *p* < .001, $\text{η}_{p}^{2}$ = .53]. And there was no significant interaction between group and response hand [*F*(1, 30) = 0.29, *p* = .60, $\text{η}_{p}^{2}$ = .01].

[Insert Figure S1 about here]

**CRT Task**

Next, we evaluated performance on the CRT task. For the left handgrip-control pair. from ANOVA of CRTs, we did not observe any significant difference in CRT between the left handgrip group and the left control group [*F*(1, 30) = 0.1, *p* = .69, $\text{η}_{p}^{2}$ = .005], or between right hand response and left hand response [*F*(1, 30) = 2.11, *p* = .16, $\text{η}_{p}^{2}$ = .07]. There was also no significant interaction between group and response hand [*F*(1, 30) = 0.16, *p* = .69, $\text{η}_{p}^{2}$ = .01].

[Insert Figure S2 about here]

Based on ANOVA of CRTs of the right handgrip-control pair, we found that there was no significant difference in CRT between the right handgrip group and the right control group [*F*(1, 30) = 0.24, *p* = .63, $\text{η}_{p}^{2}$ = .01]. There was also no significant interaction between group and response hand [*F*(1, 30) = 0.002, *p* = .96, $\text{η}_{p}^{2}$ < .001]. We observed a significant difference in SRT between right hand response and left hand response [*F*(1, 30) = 12.28, *p* = .001, $\text{η}_{p}^{2}$ = .29].

**Figure S1**

*SRT of Groups when the SRT Task was Executed under Two Different Conditions*

*Note*. Participants were asked to respond to target stimuli with the required hand, depending on the block, either with the ipsilateral or contralateral hand with reference to the intervention side (i.e., hand squeezing).

**Figure S2**

*CRT of Groups when the CRT Task was Executed under Two Different Conditions*

*Note*. Participants were asked to respond to target stimuli and distracting stimuli with the required hand, which was dependent on the block, either with the ipsilateral or contralateral hand with reference to the intervention side (i.e., hand squeezing).
