# Supplementary material for: Effects of unilateral dynamic handgrip on reaction time and error rate
Source: Cogn Process. 2022 Feb 10;23(2):169–78. doi: 10.1007/s10339-022-01080-7 (PMC9072264; doi:10.1007/s10339-022-01080-7)
Supplement: Supplementary file 3 — Supplementary file3 (DOCX 424 kb) [file 10339_2022_1080_MOESM3_ESM.docx]

Supplementary materials 3 for

**Effects of Unilateral Dynamic Handgrip on Reaction Time and Error Rate**

Arash Mirifar*, Mengkai Luan*, and Felix Ehrlenspiel

**Results**

**CRT Task**

To control for the potential effects of the intervention within the ~ 6 min (i.e., the required time for CRT task in each block), we analyzed the performance in the first 40 trials of each block in the CRT task. For the left handgrip-control pair, from ANOVA of CRTs, we did not observe any significant difference in CRT between the left handgrip group and the left control group [*F*(1, 30) = 0.027, *p* = .87, $\text{η}_{p}^{2}$ = .001], or between right hand response and left hand response [*F*(1, 30) = 3.11, *p* = .09, $\text{η}_{p}^{2}$ = .09]. There was also no significant interaction between group and response hand [*F*(1, 30) = 0.90, *p* = .35, $\text{η}_{p}^{2}$ = .03]. In addition, we found that response errors were infrequent in both the intervention and control group (6.9% and 4.8%, respectively), and ANOVA of error rates did not produce any effect, [*F*s < 1.66, *p*s > .21, $\text{η}_{p}^{2}$s < .06; see Figure S1, Figure S2].

[Insert Figure S1 about here]

[Insert Figure S2 about here]

Based on ANOVA of CRTs of the right handgrip-control pair, we found that there was no significant difference in CRT between the right handgrip group and the right control group [*F*(1, 30) = 0.58, *p* = .45, $\text{η}_{\boldsymbol{p}}^{\boldsymbol{2}}$ = .02]. There was also no significant interaction between group and response hand [*F*(1, 30) = 0.31, *p* = .58, $\text{η}_{\boldsymbol{p}}^{\boldsymbol{2}}$ = .01]. We observed a significant difference in CRT between right hand response and left hand response [*F*(1, 30) = 8.67, *p* = .006, $\text{η}_{\boldsymbol{p}}^{\boldsymbol{2}}$ = .22], with faster CRTs of the right hand response (*M* = 469 ms) than CRTs of the left hand response (*M* = 499 ms). Response errors were infrequent (9.0% and 5.9% in the intervention and control group, respectively), and ANOVA of error rates did not produce any effect [*F*s < 2.23, *p*s > .14, $\text{η}_{\boldsymbol{p}}^{\boldsymbol{2}}$s < .07; see Figure S1, Figure S2].

**Figure S1**

*CRT of Groups when the CRT Task was Executed under Two Different Conditions*

*Note*. Participants were asked to respond to target stimuli and distracting stimuli with the required hand, which was dependent on the block, either with the ipsilateral or contralateral hand with reference to the intervention side (i.e., hand squeezing). Error bars represent standard errors.

**Figure S2**

*Error Rate of Groups when the CRT Task was Executed under Two Different Conditions*

*Note*. Participants were asked to respond to target stimuli and distracting stimuli with the required hand, which was dependent on the block, either with the ipsilateral or contralateral hand with reference to the intervention side (i.e., hand squeezing). Error bars represent standard errors.
